# Extracellular Nucleotides Inhibit Insulin Receptor Signaling, Stimulate Autophagy and Control Lipoprotein Secretion

**DOI:** 10.1371/journal.pone.0036916

**Published:** 2012-05-10

**Authors:** Cynthia Chatterjee, Daniel L. Sparks

**Affiliations:** Atherosclerosis, Genetics and Cell Biology Group, University of Ottawa Heart Institute, Ottawa, Ontario, Canada; Blaise Pascal University, France

## Abstract

Hyperglycemia is associated with abnormal plasma lipoprotein metabolism and with an elevation in circulating nucleotide levels. We evaluated how extracellular nucleotides may act to perturb hepatic lipoprotein secretion. Adenosine diphosphate (ADP) (>10 µM) acts like a proteasomal inhibitor to stimulate apoB100 secretion and inhibit apoA-I secretion from human liver cells at 4 h and 24 h. ADP blocks apoA-I secretion by stimulating autophagy. The nucleotide increases cellular levels of the autophagosome marker, LC3-II, and increases co-localization of LC3 with apoA-I in punctate autophagosomes. ADP affects autophagy and apoA-I secretion through P2Y_13_. Overexpression of P2Y_13_ increases cellular LC3-II levels by ∼50% and blocks induction of apoA-I secretion. Conversely, a siRNA-induced reduction in P2Y_13_ protein expression of 50% causes a similar reduction in cellular LC3-II levels and a 3-fold stimulation in apoA-I secretion. P2Y_13_ gene silencing blocks the effects of ADP on autophagy and apoA-I secretion. A reduction in P2Y_13_ expression suppresses ERK1/2 phosphorylation, increases the phosphorylation of IR-β and protein kinase B (Akt) >3-fold, and blocks the inhibition of Akt phosphorylation by TNFα and ADP. Conversely, increasing P2Y_13_ expression significantly inhibits insulin-induced phosphorylation of insulin receptor (IR-β) and Akt, similar to that observed after treatment with ADP. Nucleotides therefore act through P2Y_13_, ERK1/2 and insulin receptor signaling to stimulate autophagy and affect hepatic lipoprotein secretion.

## Introduction

Chronic hyperglycemia in insulin resistance is known to increase the risk of cardiovascular disease and to be associated with elevated plasma apoB100 and low HDL levels [1], [2]. Elevated blood glucose is also known to stimulate nucleotide secretion and purinergic signaling [3], [4]. Under stress or injury, blood and vascular cells release nucleotides, such as ATP and ADP [5], [6]. Extracellular nucleotide concentration in the bloodstream is normally in the nM-µM range [7], [8], but can increase significantly in disease states [5], [9], [10]. Purinergic signaling events stimulate mitogen-activated protein kinase (MAPK) pathways and trigger the release of pro-inflammatory cytokines [6], [11], [12]. Extracellular nucleotides thereby directly impact the development of cardiovascular disease by promoting an “injury response” in circulating blood cells and vascular tissues [11]–[13].

Extracellular nucleotides affect hepatic lipoprotein metabolism through membrane G-protein coupled receptors (GPCR) [14], [15]. Compounds that stimulate HDL secretion from the liver appear to act through an inhibition of nucleotide signaling. Niacin has been shown to act through GPCR pathways to stimulate the secretion of HDL [16], [17] and niacin is thought to inhibit the cellular degradation of apoA-I through an inhibition of nucleotide signaling [18]. We have shown that linoleic acid phospholipids (i.e. DLPC) also act through nucleotide signaling pathways to stimulate HDL secretion [19]. These phospholipids uniquely affect MAPK and protein kinase B (Akt) signaling [20] to block apoA-I degradation in liver cells [21].

Factors that stimulate or inhibit HDL secretion from the liver appear to have the opposite effect on the secretion of the LDL protein, apoB100. ApoB100 secretion from liver cells is regulated by protein folding and proteasomal degradation [22], [23] and proteasomal inhibitors are known to stimulate the secretion of apoB100 [23]. Proteasomal inhibitors also stimulate cellular autophagic pathways [24], [25]. Autophagy is an adaptive cellular “stress response” that promotes the lysosomal degradation of cytosolic components when a cell is stimulated by stressors, i.e. nutrient deprivation, extracellular signals, hormones, cytokines and pathogens [26], [27]. Autophagy is designed to protect the cell by eliminating harmful cellular components through catabolism and recycling. Nucleotides act much like proteasomal inhibitors to stimulate apoB100 secretion and autophagy. The nucleotide, adenosine diphosphate (ADP), significantly increases apoB100 secretion from liver cells and increases the levels of the autophagy marker, microtubule-associated protein 1 light chain 3 (LC3-II). Autophagy has been shown to be associated with cardiovascular disease and studies suggest that excessive autophagy can lead to cardiac hypertrophy and heart failure [28], [29]. Pharmacological intervention to regulate cellular autophagy may therefore have therapeutic value in the treatment of cardiovascular disease.

This study shows that ADP acts through the specific GPCR, P2Y_13_, to stimulate autophagy and block HDL secretion. While stimulation in purinergic signaling would be expected to affect cellular autophagy through MAPK pathways [26], [30], we now show that ADP also acts through P2Y_13_ to block insulin receptor (IR-β) signaling and prevent the activation of Akt. The inhibition of insulin signaling pathways and Akt phosphorylation are known to stimulate autophagy [26], [27]. ADP therefore stimulates autophagy and inhibits HDL secretion by both a stimulation of MAPK and inhibition of Akt. The study suggests that elevations in circulating nucleotide levels in hyperglycemic states may affect hepatic lipoprotein secretion through a stimulation in purinergic signaling and a coordinated regulation of both proteasomal and autophagic protein degradation.

## Materials and Methods

### Reagents

Dilinoleoylphosphatidylcholine (DLPC) was obtained from Avanti Polar Lipids (Alabaster, AL). Adenosine 5′-diphosphate sodium salt (ADP), adenosine triphosphate (ATP), chloroquine diphosphate salt as well as the PI3 kinase inhibitors, 3-methyladenine (3-MA) and Wortmannin were purchased from Sigma-Aldrich (Oakville, ON). The antibody to P2Y_13_ was obtained from Abcam (Cambridge, MA). The LC3 polyclonal antibody was purchased from MBL International (Woburn, MA). Antibodies to phosphorylated ERK1/2 (p44/p42), phosphorylated Akt (Ser473), phosphorylated mTOR (Ser2448), phosphorylated IR-β (Tyr1345) and β-actin, as well as the mTOR inhibitor, rapamycin, were all obtained from Cell Signaling Technology (Danvers, MA). Human TNFα was purchased from Calbiochem (San Diego, CA). The monoclonal antibody to human apoA-I was purchased from Meridian Life Sciences, Inc (Saco, ME). The antibody to apoB (1D1) was obtained from Dr. Milne and Dr. Marcel (University of Ottawa Heart Institute). Affinity purified peroxidase linked goat anti-mouse and anti-rabbit antibodies were purchased from GE Healthcare (UK). All Stars Negative control small interference RNA (siRNA) were purchased from Qiagen (Mississauga, ON) and human P2Y_13_ siRNA were purchased from Thermo Scientific Dharmacon (Lafayette, CO). Human P2Y_13_ plasmid was purchased from Origene (Rockville, MD). Inhibitors were of analytical grade and were solubilized in dimethyl sulfoxide (DMSO).

### Cells and Cell Culture

Human hepatocarcinoma, HepG2, cells were regularly maintained in Dulbecco's modified Eagle medium (DMEM) (5 g/L glucose) containing 10% fetal bovine serum (FBS) and 1% penicillin/streptomycin. Passages 4–10 were used and cells that were 80% confluent were treated with DLPC, nucleotides and/or inhibitors for the indicated times and concentrations under serum-free conditions. Cell viability was evaluated after all treatment conditions.

### Preparation of DLPC Micelles

DLPC micelles were prepared in DMSO by sonication as previously described [20]. Purity of all phospholipids was >99%.

### Knockdown of Human P2Y_13_ by Small Interference RNA

HepG2 cells were transiently transfected with All Stars Negative control siRNA from Qiagen (Mississauga, ON) or two different P2Y_13_ siRNA sequences (ACCUUCAUCAUCUACCUCAAAUU or GACACUCAUGCUUCCUUUCAAUU) from Thermo Scientific Dharmacon (Lafayette, CO), by reverse transfection using Lipofectamine 2000 (Invitrogen, Carlsbad, CA) in 12-well plates. In brief, complexes were prepared per manufacturer's specifications with a Lipofectamine 2000-to-siRNA volume-to-mole ratio of 2∶40 (µL∶ρmol) in 200 µL of Opti-MEM I Reduced Serum Media (Invitrogen, Carlsbad, CA). Lipofectamine-siRNA complexes were added to the cells immediately after the cells were seeded at a density of 500,000 cells/well in a volume of 1 mL of normal growth media containing 10% FBS in the absence of penicillin/streptomycin. The cells were treated with ADP, TNFα or DLPC in serum-free DMEM 48 h after transfection. Cell media and lysate samples were harvested at the indicated timepoints for both immunoblot and ELISA analysis. Transfection of the control and test siRNA caused no cytotoxic effects.

### Overexpression of Human P2Y_13_ by Plasmid

The pCMV6 vector containing the full-length human P2Y_13_ cDNA was purchased from Origene (Rockville, MD). HepG2 cells were transiently transfected with control plasmid or the pCMV6-P2Y_13_ plasmid by reverse transfection using FuGENE HD (Roche Applied Science, Laval, QC). Complexes were prepared per manufacturer's instructions with a FuGENE HD-to-DNA volume-to-mass ratio of 6∶2 (µl to µg) in 100 µL of Opti-MEM I Reduced Serum Media (Invitrogen, Carlsbad, CA). HepG2 cells were trypsinized and seeded in 12-well plates at a density of 500,000 cells/well in a volume of 1 mL in normal growth media containing 10% FBS in the absence of penicillin/streptomycin and then 50 µL of the transfection complexes were immediately added to the suspended cells. The cells were treated with ADP, TNFα or DLPC in serum-free DMEM 48 h after transfection. Cell media and lysate samples were harvested at the indicated timepoints for both immunoblot and ELISA analysis. Transfection of the control and test plasmid caused no cytotoxic effects.

### ApoA-I ELISA

ApoA-I concentration in conditioned media and cell lysate samples were analyzed by ELISA according to manufacturer's instructions as previously described [20]. 96-well plates were coated overnight with a mouse anti-human apoA-I monoclonal antibody (Meridian Life Sciences, Inc, Saco, ME). Wells were blocked with BSA and then samples/standards were incubated in the wells for 2 h, followed by a 1 h incubation with a horseradish peroxidase-linked goat anti-human apoA-I antibody. K-blue Max TMB substrate (Neogen, Inc) was added to each well, the reaction was stopped with 0.2 N HCl, and the absorbance was recorded at 450 nm. ApoA-I concentration in the conditioned media and cell lysate samples were normalized to total cell protein.

### Immunoblot Analysis

After treatment for the indicated timepoints, cells were washed twice with ice-cold PBS. Cells were lysed in NP-40 lysis buffer (Biosource, Camarillo, CA) supplemented with 1 mM PMSF and 1× protease inhibitor cocktail (Sigma, Saint Louis, MO). Cell protein concentrations were determined by the BCA Protein Assay (Thermo Fisher Scientific, Waltham, MA). Cell lysate samples containing equal total protein (30 µg) were separated by SDS-PAGE and analyzed by Western blot using specific antibodies to apoA-I, apoB100, P2Y_13_, LC3, p62, p-ERK1/2, p-Akt, p-mTOR, p-IR-β, and β-actin. Blots were exposed using the Alpha Innotech FluorChem™ HD Imager and band intensities were quantified with the Alpha Ease FC™ software.

### Immunofluorescence and Colocalization

After treatment, the cells were fixed with 4% paraformaldehyde, permeabilized with 0.1% Triton X-100, and then blocked in 10% FBS for 30 min. The cells were then immunostained with 1∶200 rabbit polyclonal anti-LC3 (MBL International, Woburn, MA) and 1∶200 mouse anti-apoA-I antibodies (Meridian Life Sciences, Saco, ME) for 1 h and then with 1∶1000 Alexa Fluor 488 goat anti-rabbit IgG (H+L) and 1∶1000 Alexa Fluor 647 donkey anti-mouse IgG (H+L) (Invitrogen, Burlington, ON) for an additional 1 h. Images were acquired on an Olympus 1X80FV1000 confocal laser microscope using Olympus Fluoview FV1000 software and colocalization was quantified using FV10-ASW V2.1.

### Statistical Analysis

Values are shown as Mean ± SD for at least 3 independent experiments and *P*<0.05 was considered significant. Differences between mean values were evaluated by one-way analysis of variance (ANOVA) on ranks by a pairwise multiple comparison using the Student-Newman-Keuls post-hoc test (SigmaStat; Systat Software, Inc., San Jose, CA).

## Results

### Extracellular nucleotides inhibit hepatic apoA-I secretion

**Figure 1:**
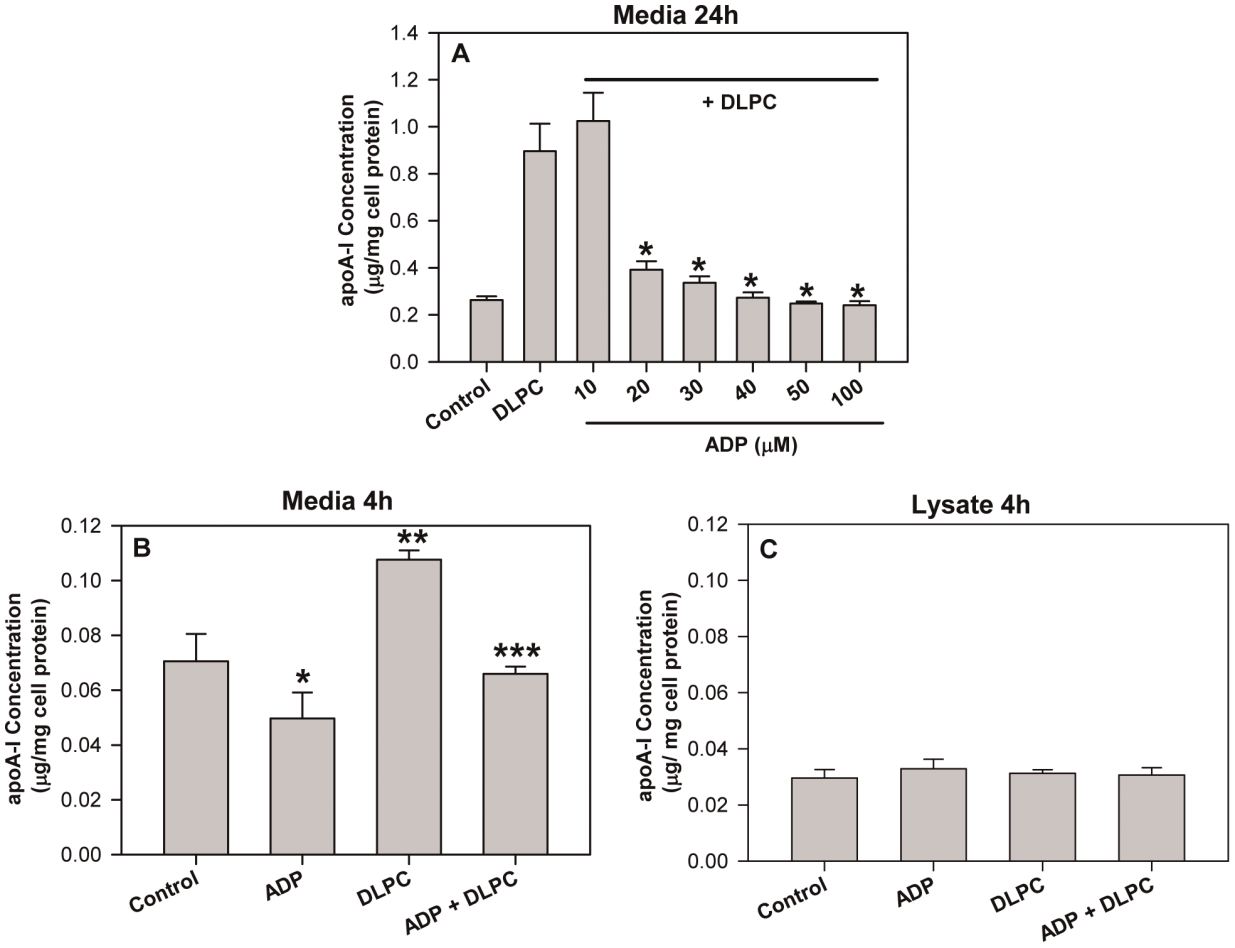
Extracellular nucleotides block the induction of apoA-I secretion. HepG2 cells were pre-treated with adenosine diphosphate (ADP) (10 to 100 µM)) for 30 min. and then incubated with 12 µM DLPC in serum-free DMEM media. (**A**) Conditioned media was collected after 24 h and apoA-I concentration was quantified by ELISA. ApoA-I concentration in the media is normalized to total cell protein and expressed as mean ± SD of 3 independent experiments. *P<0.05 vs DLPC. (**B**) Conditioned media was collected after 4 h treatment with 100 µM ADP +/− DLPC and apoA-I concentration was quantified by ELISA. ApoA-I concentration in the media is normalized to total cell protein and expressed as mean ± SD of 3 independent experiments. *P<0.01 vs ADP, **P<0.001 vs Control, ***P<0.001 vs DLPC. (**C**) Cell lysates were collected after 4 h of treatment and apoA-I concentration was quantified by ELISA. ApoA-I concentration in the cell lysate is normalized to total cell protein and expressed as mean ± SD of 3 independent experiments.

Previous work has shown that membrane ATPases control the nucleotide-dependent endocytosis of HDL [14], [31] and that inhibition of ADP production by membrane ATPases may promote HDL secretion [18], [19]. To determine if ADP directly affects hepatic HDL secretion, experiments were undertaken to evaluate the effect of ADP on apoA-I secretion from liver cells. As we have previously shown [19], dilinoleoylphosphatidylcholine (DLPC) (12 µM) stimulates a ∼4-fold increase in apoA-I secretion (accumulation in the media) from HepG2 cells over 24 h (**Figure 1A**). Pre-treatment of the cells with 20–100 µM ADP completely blocked the induction of apoA-I secretion by DLPC (**Figure 1A**). Treatment with ATP (100 µM) had a lesser effect than ADP and only blocked the induction of apoA-I secretion by 60% (**Figure S1**). The effects of DLPC and ADP on apoA-I secretion were also evident after short time periods. ADP (100 µM) was able to significantly reduce basal apoA-I secretion at 4 h (**Figures 1B** **and** **2C**). DLPC stimulated the secretion of apoA-I after 4 h and ADP completely blocked this DLPC-induction of apoA-I secretion. Lower doses of ADP (25 and 50 µM) also inhibited apoA-I secretion at 4 h (not shown). ADP treatment had no effect on cellular apoA-I levels after 4 h (**Figure 1C**) or 24 h (not shown). ADP therefore significantly reduced apoA-I mass (media+lysate) at 4 h by 20% (ADP alone) to 30% (ADP+DLPC).

**Figure 2:**
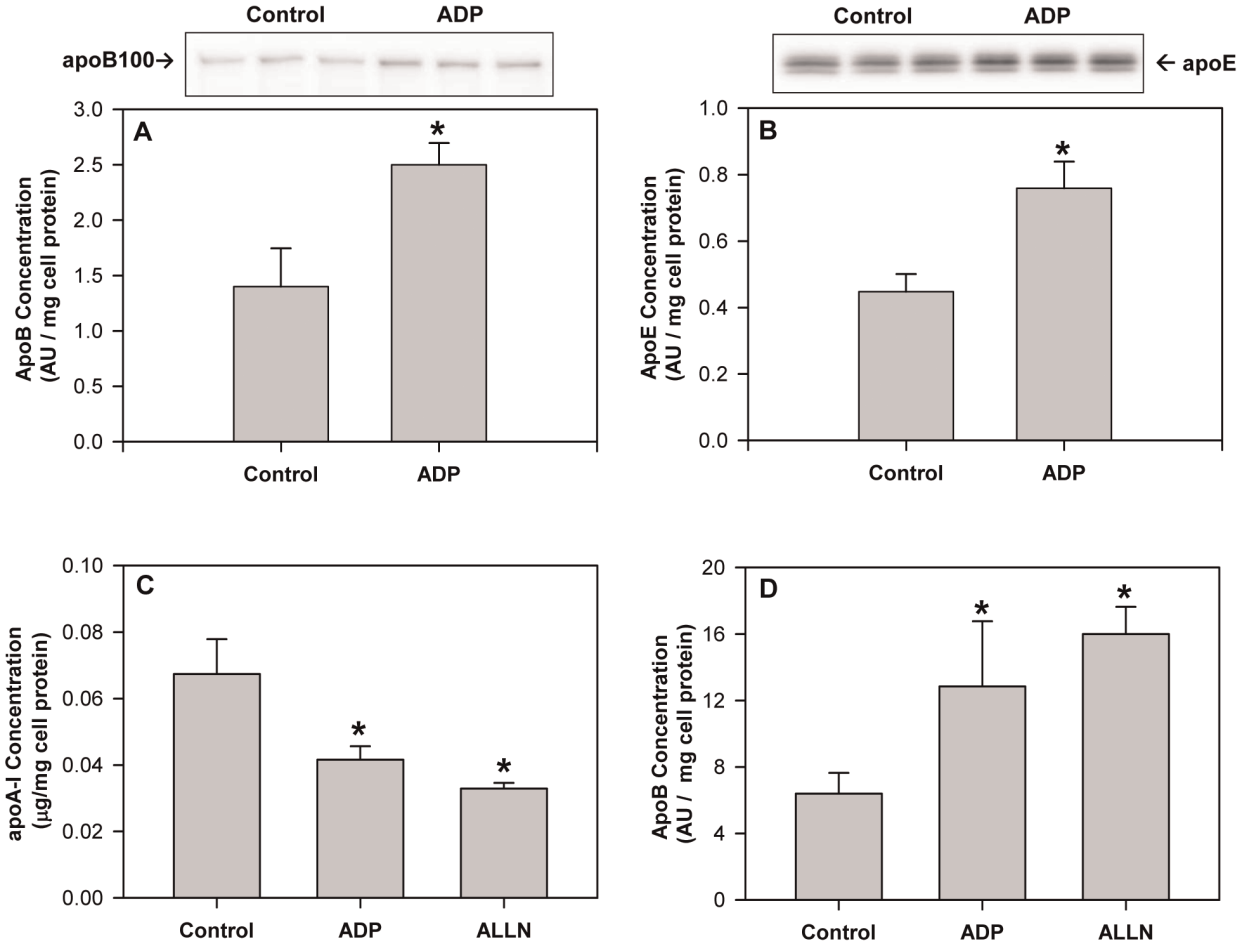
ADP stimulates apoB100 and apoE secretion. (**A&B**) HepG2 cells were incubated with 100 µM adenosine diphosphate (ADP) for 24 h in serum-free DMEM media. Conditioned media was collected and immunoblotted for apoB100 (**A**) and apoE (**B**). Histograms represent band densitometry analysis of apoB100 or apoE, normalized to total cell protein and expressed as mean ± SD of 3 independent experiments. *P<0.01 vs Control. (**C&D**) HepG2 cells were treated with 100 µM adenosine diphosphate (ADP) or 25 µM ALLN (N-Acetyl-L-leucyl-L-leucyl-L-norleucinal) for 4 h in serum-free DMEM media. (**C**) Conditioned media was collected and apoA-I concentration was quantified by ELISA. ApoA-I concentration in the media is normalized to total cell protein and expressed as mean ± SD of 3 independent experiments. *P<0.05 vs Control. (**D**) ApoB100 concentration in the media was determined by Western blot and histograms represent band densitometry analysis of apoB100, normalized to total cell protein and expressed as mean ± SD of 3 independent experiments. *P<0.05 vs Control.

### Adenosine diphosphate stimulates hepatic apoB100 and apoE secretion

Experiments were undertaken to determine if ADP might affect the secretion of other apolipoproteins. In contrast to that observed with apoA-I, ADP significantly increased apoB100 secretion (**Figure 2A**) and apoE secretion (**Figure 2B**) from HepG2 cells after 24 h. ApoB100 secretion is regulated by proteasomal degradative pathways [22], [23], [32] and therefore the effect of ADP on apolipoprotein secretion was compared to that of proteasomal inhibitors. ADP and ALLN (25 µM) decreased apoA-I levels in the media to a similar extent at 4 h (**Figure 2C**), but increased the concentration of apoB100 in the media (**Figure 2D**). The work suggests that ADP may regulate apolipoprotein secretion in a similar fashion to that observed with proteasomal inhibitors.

### Adenosine diphosphate stimulates autophagy

**Figure 3:**
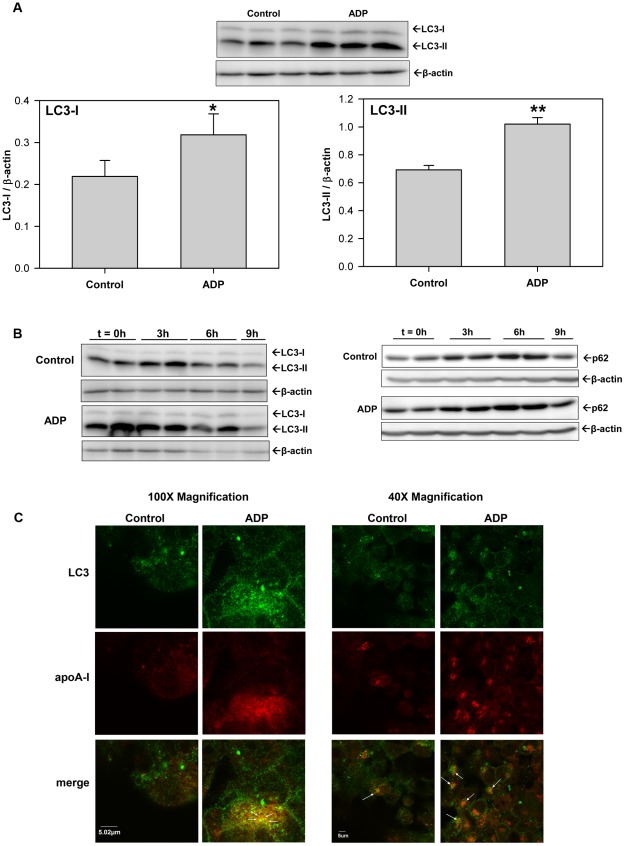
ADP stimulates autophagy and increases cellular LC3-II. (**A**) HepG2 cells were treated with 100 µM adenosine diphosphate (ADP) for 4 h in serum-free DMEM media. Cell lysates were immunoblotted for LC3. Histograms represent band densitometry analysis of LC3-I and LC3-II, normalized to β-actin and expressed as mean ± SD of 3 independent experiments. *P<0.05 vs Control, **P<0.001 vs Control. (**B**) HepG2 cells were serum-starved (Control) or pretreated for 30 min. with 100 µM adenosine diphosphate (ADP) in serum-free DMEM media and then lysates were harvested at the indicated timepoints (0, 3, 6 & 9 h). (**Left panels**) Cell lysates were immunoblotted for LC3 and β-actin. (**Right panels**) Cell lysates were immunoblotted for p62 and β-actin. Blots are representative of 2 independent experiments. (**C**) HepG2 cells were serum-starved (Control) or treated with 100 µM ADP in serum-free DMEM media for 4 h. Cells were fixed and permeabilized and then LC3 and apoA-I were detected by indirect immunofluorescence using primary antibodies against human LC3 and apoA-I, and Alexa Fluor-conjugated secondary antibodies (Alexa Fluor 488 goat anti-rabbit Ab (green for LC3) and Alexa Fluor 647 anti-mouse Ab (red for apoA-I)) by confocal microscopy. Micrograph 100× and 40× images of representative cells from 2 independent experiments done in quadruplicate are shown.

Studies have shown that proteasomal inhibitors also act to stimulate autophagy [24], [25] and therefore experiments were undertaken to determine if ADP affects autophagy and impacts the level of the autophagy marker, LC3. HepG2 cells were treated with 100 µM ADP for 4 h and then cell lysates were probed for LC3. **Figure 3A** shows that ADP significantly increased both LC3-I and LC3-II levels at 4 h. The data suggests that ADP stimulates autophagy and this view is confirmed by the data in **Figure 3B**. To determine if ADP uniquely affects the activation versus flux of LC3 in HepG2 cells, 9 h time course studies were undertaken. **Figure 3B** shows that serum starvation (**control**) increases LC3-II levels in HepG2 cell lysates at 3 h, which return to below basal levels by 9 h (**left panel blots**). ADP increases LC3-II levels more quickly than the control starvation (within the 30 min pretreatment) and maintains increased cellular LC3-II levels for 6 h (**Figure 3B, left panel blots**). Similar results were seen with another autophagy marker protein, p62 (**Figure 3B, right panel blots**).

Immunofluorescent experiments using laser confocal microscopy confirm the Western blot data and further showed that apoA-I and LC3 are colocalized within autophagosomes. **Figure 3C** (**and Figure S2**) illustrates 4 h confocal micrographic images of indirect immunofluorescent stained apoA-I and LC3 in fixed and permeabilized HepG2 cells. Control starvation conditions show the staining pattern for both LC3 (green) and apoA-I (red) and illustrate low levels of colocalization of the two proteins in the merged images (yellow-orange). Similar to that shown in **Figures 3A&B**, ADP increases cellular LC3 levels relative to control, and confocal images show LC3 to be localized to punctate autophagosomal structures within the cell (**Figure 3C**). When LC3 and apoA-I immunofluorescent images for ADP treated cells are merged, the images show significantly (P<0.001) more yellow-orange structures in ADP-treated vs control cells (23.8%±5.2% vs 7.5%±3.0%), which indicates that ADP significantly increases the colocalization of apoA-I with LC3. ADP-dependent increases in LC3-II levels were completely inhibited by treatment with 3-methyladenine (3-MA) (5 mM) or U0126 (10 µM), while LC3-II levels were further increased by treatment with chloroquine (50 µM) or Wortmannin (10 µM) (**Figure S3**).

### P2Y_13_ expression affects autophagy and apoA-I secretion

**Figure 4:**
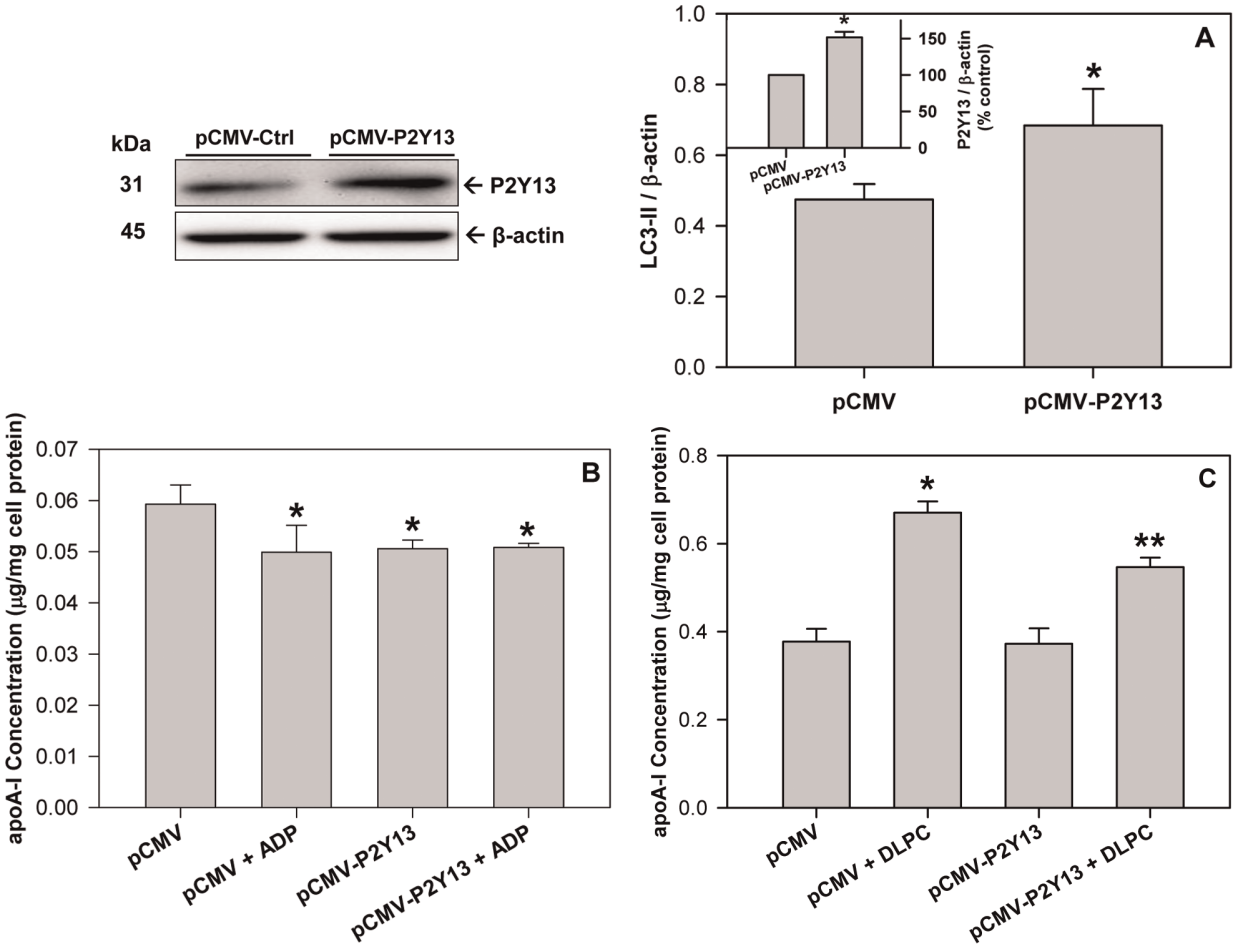
Increasing P2Y_13_ expression stimulates autophagy and blocks apoA-I secretion. HepG2 cells were transfected with either a control pCMV plasmid (pCMV) or a pCMV plasmid expressing human P2Y_13_ (pCMV-P2Y13). Cell lysates were collected 48 h after transfection and immunoblotted for P2Y_13_ to measure protein overexpression (**Upper left panel**). Histograms represent band densitometry analysis of P2Y_13_, normalized to β-actin (**inset A**) and expressed as mean ± SD of 3 independent experiments. *P<0.05 vs pCMV. (**A**) Cell lysates were immunoblotted for LC3 and histograms represent band densitometry analysis of LC3-II, normalized to β-actin and expressed as mean ± SD of 3 independent experiments. *P<0.05 vs pCMV. (**B**) Transfected cells were treated with 100 µM ADP in serum-free DMEM media for 4 h, conditioned media was collected and apoA-I concentration was quantified by ELISA. ApoA-I concentration in the media is normalized to total cell protein and expressed as mean ± SD of 3 independent experiments. *P<0.05 vs pCMV Control. (**C**) Transfected cells were treated with 12 µM DLPC in serum-free DMEM media for 24 h, conditioned media was collected and apoA-I concentration was quantified by ELISA. ApoA-I concentration in the media is normalized to total cell protein and expressed as mean ± SD of 3 independent experiments. *P<0.01 vs pCMV Control, **P<0.01 vs pCMV+DLPC.

Extracellular ADP affects cellular apoA-I metabolism through the GPCR, P2Y_13_ [15] in hepatocytes and therefore the effect of P2Y_13_ expression on autophagy and apoA-I secretion were evaluated. Transfecting HepG2 cells with a pCMV6-P2Y_13_ plasmid promoted a ∼50% increase in P2Y_13_ expression (**Figure 4** **, Western blot and inset**) and a parallel increase in LC3-II levels (**Figure 4A**). Treatment of liver cells with a pCMV6-P2Y_13_ plasmid blocked the basal secretion of apoA-I at 4 h, similar to that observed after treatment with ADP, while a combination of pCMV6-P2Y_13_ and ADP had no additional effect on apoA-I secretion (**Figure 4B**). Increasing P2Y_13_ expression in the liver cells significantly reduced the DLPC-induction of apoA-I secretion after 24 h (**Figure 4C**).

**Figure 5:**
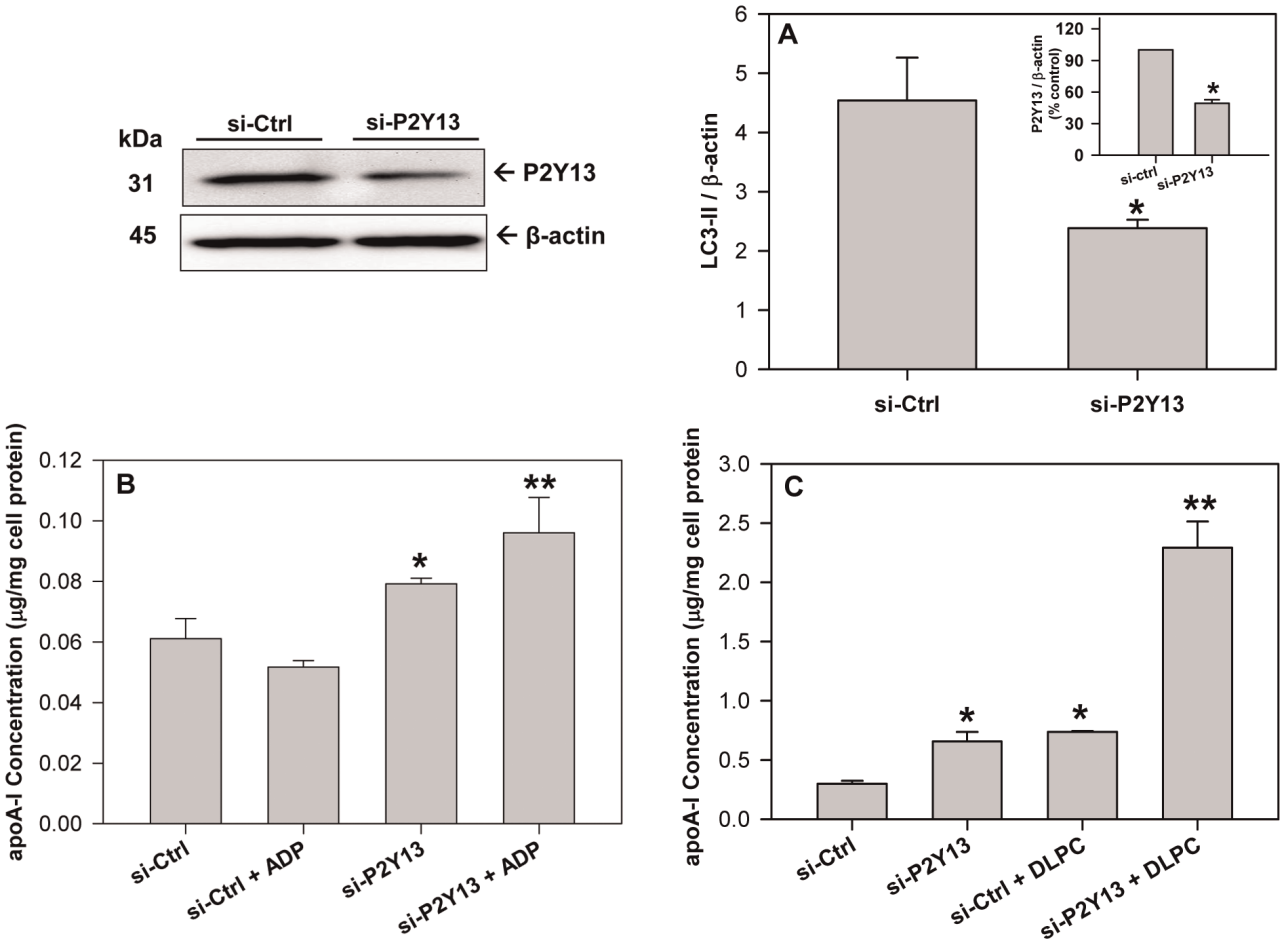
Reducing P2Y_13_ expression blocks autophagy and stimulates apoA-I secretion. HepG2 cells were transfected with either a negative control (si-ctrl) or a siRNA against human P2Y_13_. Cell lysates were collected 48 h after transfection and immunoblotted for P2Y_13_ to confirm protein knockdown (**Upper left panel**). (**A**) Cell lysates were immunoblotted for LC3 and histograms represent band densitometry analysis of LC3-II, normalized to β-actin, and expressed as mean ± SD of 3 independent experiments. *P<0.05 vs si-Ctrl. (**Inset A**) Histograms represent band densitometry analysis of P2Y_13_ normalized to β-actin and expressed as mean ± SD of 3 independent experiments. *P<0.05 vs si-Ctrl. (**B**) Transfected cells were treated with 100 µM ADP in serum-free DMEM media for 4 h, conditioned media was collected and apoA-I concentration was quantified by ELISA. ApoA-I concentration in the media is normalized to total cell protein and expressed as mean ± SD of 3 independent experiments. *P<0.05 vs si-Ctrl, **P<0.05 vs si-P2Y13 (**C**) Transfected cells were treated with 12 µM DLPC in serum-free DMEM media for 24 h, conditioned media was collected and apoA-I concentration was quantified by ELISA. ApoA-I concentration in the media is normalized to total cell protein and expressed as mean ± SD of 3 independent experiments. *P<0.01 vs si-Ctrl, **P<0.001 vs si-Ctrl+DLPC.

Reducing P2Y_13_ expression had the opposite effect. Transfecting HepG2 cells with P2Y_13_ siRNA promoted a >50% reduction in P2Y_13_ protein expression (**Figure 5** **, Western blot and inset**) and caused a similar reduction in LC3-II levels (**Figure 5A**). P2Y_13_ siRNA significantly stimulated apoA-I secretion at both 4 h and 24 h (**Figures 5B and 5C**). After a 4 h incubation, ADP was unable to block apoA-I secretion in cells treated with P2Y_13_ siRNA, but conversely, increased apoA-I secretion relative to P2Y_13_ siRNA treatment alone (**Figure 5B**). Treatment with DLPC (12 µM) or P2Y_13_ siRNA for 24 h stimulated apoA-I secretion by ∼2.5-fold, while treatment with both DLPC and P2Y_13_ siRNA promoted a ∼10-fold stimulation in apoA-I secretion from HepG2 cells (**Figure 5C**). P2Y_13_ siRNA completely blocked the effect of ADP on LC3-II (**Figure S4**).

### Nucleotides and P2Y_13_ regulate MAPK signaling

**Figure 6:**
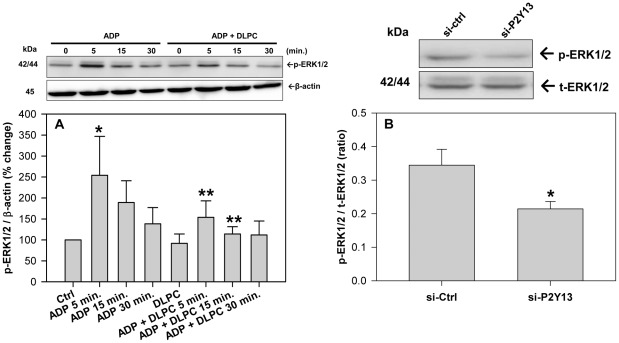
Extracellular nucleotides and P2Y_13_ expression regulate ERK1/2 signaling. (**A**) HepG2 cells were pre-treated with 12 µM DLPC for 30 min. and then incubated with and without ADP (100 µM) for 0, 5, 15 and 30 min in DMEM serum-free media. Cell lysates were immunoblotted for phosphorylated ERK1/2. Histograms represent densitometry analysis of p-ERK1/2 normalized to β-actin and expressed as mean percent change ± SD of 3 independent experiments. *P<0.01 vs Ctrl, **P<0.05 vs ADP 5 min. (**B**) HepG2 cells were transfected with either negative control (si-Ctrl) or P2Y_13_ siRNA (si-P2Y_13_) and incubated for 24 h. Cell lysates were immunoblotted for phosphorylated and total ERK1/2. Histograms represent band densitometry analysis of the ratio of phospho-ERK1/2 (p-ERK1/2) to total ERK1/2 (t-ERK1/2) and are expressed as mean ± SD for 3 independent experiments. *P<0.05 vs. control siRNA.

Cellular autophagy is regulated by the activation of MAPK (ERK1/2) and therefore the importance of ERK1/2 in ADP-dependent purinergic signaling was investigated. The nucleotide, ADP, stimulates ERK1/2 phosphorylation over a 30 min period and DLPC blocked the activation of ERK1/2 by ADP (**Figure 6A**). P2Y_13_ expression also affects ERK1/2 phosphorylation and a 50% reduction in cellular P2Y_13_ expression caused a significant reduction in ERK1/2 phosphorylation after 24 h (**Figure 6B**). Cell viability was unaffected by the P2Y_13_ siRNA.

### Nucleotides and P2Y_13_ regulate insulin receptor signaling

**Figure 7:**
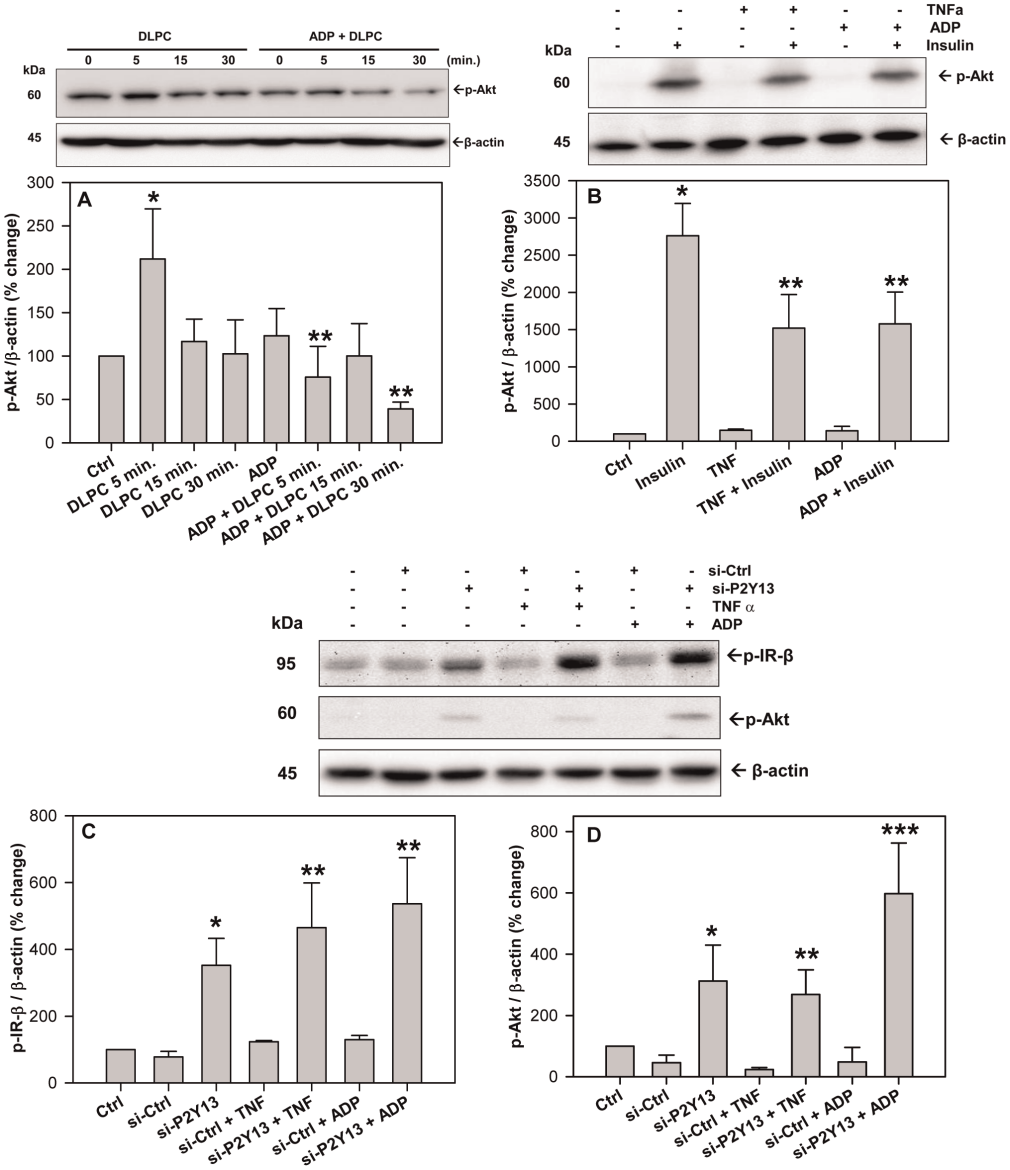
Extracellular nucleotides and P2Y_13_ expression regulate insulin receptor signaling. (**A**) HepG2 cells were pre-treated with 12 µM DLPC for 30 min. and then incubated with and without ADP (100 µM) for 0, 5, 15 and 30 min in DMEM serum-free media. Cell lysates were immunoblotted for phosphorylated Akt (Ser473). Histograms represent densitometry analysis of p-Akt normalized to β-actin and expressed as mean percent change ± SD of 3 independent experiments. *P<0.01 vs Control, **P<0.001 vs DLPC 5 min. (**B**) HepG2 cells were pre-treated with adenosine diphosphate (ADP) (100 µM) or TNFα (10 ng/ml) for 5 min. and then with human insulin (100 nM) for 5 min in DMEM serum-free media. Cell lysates were immunoblotted for phosphorylated Akt (Ser473). Histograms represent densitometry analysis of p-Akt normalized to β-actin and expressed as mean ± SD for 3 independent experiments. *P<0.001 vs Ctrl, **P<0.001 vs. insulin alone. (**C&D**) HepG2 cells were transfected with either negative control (si-Ctrl) or P2Y_13_ siRNA (si-P2Y_13_) and incubated for 48 h. Cells were then treated with adenosine diphosphate (ADP) (100 µM) or TNFα (10 ng/ml) for 5 min. in DMEM serum-free media. (**C**) Cell lysates were immunoblotted for phosphorylated insulin receptor (IR-β) (Tyr1345). Histograms represent densitometry analysis of p-IR-β normalized to β-actin and expressed as mean ± SD for 3 independent experiments. *P<0.01 vs si-Ctrl, ** P<0.001 vs si-Ctrl. (**D**) Cell lysates were also immunoblotted for phosphorylated Akt (Ser473). Histograms represent densitometry analysis of p-Akt normalized to β-actin and expressed as mean ± SD for 3 independent experiments.*P<0.01 vs si-Ctrl, **P<0.05 vs. si-Ctrl, ***P<0.001 vs si-Ctrl.

Cellular autophagy is activated by the inhibition of protein kinase B (Akt) [27]. DLPC stimulates the phosphorylation of Akt at 5 min. and ADP completely blocks the activation of Akt by DLPC (**Figure 7A**). ADP also inhibits the activation of Akt by insulin. **Figure 7B** shows that insulin stimulates Akt phosphorylation in HepG2 cells and that both tumor necrosis factor α (TNFα) and ADP inhibit insulin-induced Akt phosphorylation by ∼50%. Reducing P2Y_13_ expression by treatment with P2Y_13_ siRNA appeared to inhibit cellular autophagic pathways by stimulating the phosphorylation of Akt. A reduction in cellular P2Y_13_ expression significantly increases the phosphorylation of IR-β and Akt (**Figure 7C&D**) by >3-fold. P2Y_13_ gene silencing significantly augmented the insulin-induced phosphorylation of Akt (**Figure S5**) and blocked the inhibitory effect of TNFα and ADP on IR-β and Akt phosphorylation (**Figure 7C&D** **and S5**). Similar results were observed for the insulin-like growth factor receptor (IGF-1R) (not shown). P2Y_13_ overexpression had the opposite effect. Transfection of HepG2 cells with pCMV6-P2Y_13_ plasmid significantly reduced the phosphorylation of IR-β (**Figure S6**). Increasing P2Y_13_ expression significantly inhibited the insulin-induced phosphorylation of IR-β and Akt (**Figure S6**), similar to that observed after treatment of HepG2 cells with ADP (**Figure 6C**). P2Y_13_ expression significantly affected Akt phosphorylation, but had no effect on mTOR (**Figure S7**). ADP actually increased mTOR phosphorylation, while the mTOR inhibitor, rapamycin, significantly reduced p-mTOR levels (**Figure S8**).

## Discussion

Insulin resistance and hyperglycemia have been shown to perturb plasma lipoprotein metabolism and increase apoB100 levels, but decrease HDL [1], [2]. Insulin resistance is consequently a well accepted risk factor for the development of cardiovascular disease [1]. High blood glucose levels stimulate ATP production and promote the release of nucleotides from circulating blood cells, endothelial cells and smooth muscle cells [3], [4]. ATP is unstable in the circulation and is quickly converted to ADP [7], [8]. Elevations in blood nucleotide levels can impact cardiovascular disease [4], [5], [13] and inhibition of ADP-dependent thrombosis with P2Y_12_ receptor inhibitors has already shown significant cardiovascular therapeutic value [33], [34]. Niacin has also been shown to have cardiovascular therapeutic value and this molecule appears to act through another G-protein coupled receptor [16] to block purinergic signaling [18] and atherogenesis [35].

Our studies show that HDL secretion is regulated similarly in primary human hepatocytes and HepG2 liver cells and that the linoleic acid phospholipid, dilinoleoylphosphatidylcholine (DLPC), can stimulate HDL/apoA-I secretion [19], [20], [36]. DLPC appears to act much like niacin to prevent purinergic signaling by inhibiting F1-ATPase and blocking the production of ADP [19]. This view has been confirmed by the present work, which shows that ADP is a potent antagonist to the induction of apoA-I secretion by DLPC. **Figure 1** shows that an [ADP] >10 µM completely blocked the induction of apoA-I secretion by DLPC at 24 h and also at 4 h. Conversely, ADP can directly stimulate apoB100 and apoE secretion from human liver cells at 24 h (**Figure 2A&B**). The normal physiological concentration of ADP in the bloodstream has been reported to be ∼15 µM [**8**], but elevated blood glucose levels can increase nucleotide secretion and accumulation in the circulation [3], [4]. High circulatory nucleotide levels would therefore be expected to block hepatic apoA-I secretion and stimulate apoB100 output. This is indeed similar to what is thought to occur in hyperglycemic, insulin resistant patients [1], [37], [38]. This may suggest that elevations in blood nucleotide levels may be partly causative to abnormal plasma lipoprotein levels.

Since apoB100 secretion is known to be regulated by proteasomal degradation, we evaluated the effect of proteasomal inhibitors on both apoB100 and apoA-I secretion. ADP and the proteasomal inhibitor, ALLN, appear very similar and both inhibit apoA-I secretion, but stimulate apoB100 secretion at 4 h (**Figure 2C&D**). ADP may therefore act similar to proteasomal inhibitors to stimulate autophagy [24], [25]. ADP stimulates autophagy in HepG2 cells and increases the level of autophagic markers, LC3-I and LC3-II, and p62 over a 6 h period (**Figure 3**). The ability of ADP to stimulate apoB100 secretion (**Figure 2**) and increase p62 levels may indicate that ADP blocks proteasomal degradation, since p62 levels are known to rise when the proteasome is inhibited [26], [27]. Confocal studies confirmed the higher levels of LC3 after treatment with ADP. Micrographs showed that LC3-II was located in punctate autophagosomes within the liver cells and clearly showed that higher levels of LC3 and apoA-I were colocalized within autophagosomes in ADP treated cells (**Figure 3C**). The view that a stimulation in autophagy may inhibit apoA-I secretion is consistent with earlier work, which has shown that serum deprivation, a treatment well-known to directly stimulate autophagy (**Figure 3B** and [26], [27]), also inhibits apoA-I secretion of HepG2 cells by ∼50% over the first hour [**39**].

Human liver cells contain two ADP-receptors, P2Y_1_ and P2Y_13_, but HDL metabolism is primarily affected by P2Y_13_ [15], [40], [41]. ADP is a potent agonist to P2Y_13_ and stimulates a rapid (10 min) endocytic recycling pathway for extracellular apoA-I [14], [15], [31]. This recycling pathway has been shown contribute to apoA-I lipidation, cholesterol efflux and apoE resecretion, but does not promote significant apoA-I degradation [42]–[44]. Conversely, it is known that apoA-I secretion is affected by cellular degradation [18], [21] and activation of P2Y_13_ appears to stimulate degradation pathways. The direct effect of P2Y_13_ on both autophagy and apoA-I secretion from human liver cells is clearly illustrated by increasing or silencing P2Y_13_ gene expression. P2Y_13_ overexpression significantly increased cellular LC3-II levels and decreased apoA-I secretion (**Figure 4**), similar to that seen with exogenous ADP. Conversely, P2Y_13_ gene silencing with siRNA significantly decreased LC3-II levels, increased both basal apoA-I secretion and the DLPC induction in apoA-I secretion, and blocked the effects of ADP on autophagy and apoA-I secretion (**Figure S4 and** **Figure 5**). P2Y_13_ expression in HepG2 cells therefore appears to regulate apoA-I secretion through cellular autophagic pathways.

In contrast to this work, studies in P2Y_13_-deficient mice have shown that reducing P2Y_13_ expression caused a small reduction in plasma HDL levels *in vivo* [45], [46]. This may be partly due to the fact that mice are not a human equivalent model for the study of HDL metabolism, since numerous liver-specific signaling pathways differ in mice [47]. Nucleotide signaling also differs significantly in rodents [40], [48]. In studies with rat hepatocytes, ADP acts through P2Y_2_ receptors to increase [IP3] and [Ca^2+^] [48]. This does not occur in human cells. Treatment of both primary human liver cells and HepG2 cells with ADP or UDP has no effect on cellular [IP3] and [Ca^2+^] [40]. In human liver cells, ADP stimulates MAPK and reduces [cAMP] [15], [41], both of which are known to reflect activation of P2Y_13_ [49], [50]. We show that ADP stimulates ERK1/2 in human liver cells (**Figure 6A**), but ADP has the opposite effect in murine pancreatic cells and inhibits ERK1/2 phosphorylation [51]. ADP and P2Y_13_-dependent signaling may therefore be very different in humans and rodents and this may explain why P2Y_13_-deficient mice show no major lipoprotein phenotype [45], [46].

Nucleotide signaling through P2Y receptors is well known to activate MAPK pathways [49], [50] and MAPK is a well-known activator of cellular autophagy [26]. DLPC may therefore stimulate apoA-I secretion by blocking MAPK activation and preventing the autophagic degradation of apoA-I. DLPC can block an ADP-dependent activation of ERK1/2 in HepG2 cells (**Figure 6A**), similar to that shown in neuronal cells, where DLPC blocked ERK1/2 activation by TNFα and hydrogen peroxide [52]. Treatment of liver cells with the MEK1/2 inhibitor, U0126, completely blocked the ADP-dependent increase in LC3-II levels (**Figure S3**) and P2Y_13_-gene silencing also muted ERK1/2 activation (**Figure 6B**). Our data suggests that MAPK is not alone in regulating autophagy and apoA-I secretion. ADP can block the activation of Akt by DLPC and insulin (**Figure 7A&B**) and Akt is an established inhibitor of autophagy [26], [30]. Therefore, while nucleotides may affect cellular autophagic pathways through MAPK pathways, the insulin signaling-dependent Akt pathways may also play an important role in the purinergic regulation of autophagy and HDL secretion. Akt can inhibit autophagy directly, and indirectly through mTOR [26]. Our work, however suggests that ADP stimulates autophagy through mTOR-independent pathways, since P2Y_13_ expression had no effect on mTOR phosphorylation, while ADP increased p-mTOR levels (**Figures S7&S8**).

If ADP-dependent signaling through P2Y_13_ impacts Akt-dependent pathways, it follows that P2Y_13_ expression would be expected to affect insulin receptor signaling. Consistent with this view, a reduction in P2Y_13_ expression directly stimulates the phosphorylation of IR-β and Akt by >3-fold (**Figure 7**), increases insulin-dependent signaling (**Figure S5**) and completely blocks the inhibition of insulin receptor signaling by TNFα and ADP (**Figure 7**). Increasing P2Y_13_ expression has the opposite effect and significantly inhibits insulin-induced phosphorylation of IR-β and Akt (**Figure S6**). Nucleotide signaling through P2Y_13_ may therefore affect insulin receptor signaling. This work appears consistent with other studies showing that ADP acts through P2Y_13_ to inhibit insulin secretion from pancreatic beta cells [51], [53].

**Figure 8:**
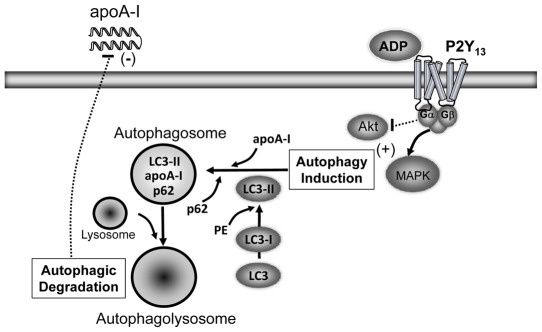
Extracellular nucleotides act through P2Y_13_ to stimulate autophagy. Elevations in blood glucose promote the secretion and accumulation of nucleotides in the circulation. Nucleotides act through P2Y_13_ to activate mitogenic pathways, inhibit insulin receptor signaling and stimulate autophagic protein degradation. Enhanced purinergic signaling in insulin resistance may give rise to a chronic induction of cellular autophagy and a reduction in apoA-I secretion from the liver.

This work shows that lipoprotein secretion and insulin signaling pathways are affected by hepatic membrane purinergic receptor signaling. Elevations in blood glucose promote the synthesis and secretion of nucleotides from circulating blood cells and vascular tissues. Nucleotides that accumulate in the circulation can then act through purinergic receptors, i.e. P2Y_13_, to stimulate mitogenic pathways and inhibit insulin receptor signaling. Enhanced purinergic signaling in insulin resistance may give rise to an inhibition of proteasomal degradation and a chronic induction of cellular autophagy (**Figure 8**). The net result is a stimulation of apoB100 secretion from the liver and a reduction in apoA-I secretion. This may partly explain the well-described lipoprotein phenotype associated with insulin resistance [1].

## Supporting Information

Figure S1 **ATP decrease apoA-I levels in the media at 24 h.** (**A**) HepG2 cells were pre-treated with adenosine diphosphate (ADP) or adenosine triphosphate (ATP) (100 µM) for 30 min. and then incubated with 12 µM DLPC in serum-free DMEM media. Conditioned media was collected after 24 h treatment and apoA-I concentration was quantified by ELISA. ApoA-I concentration in the media is normalized to total cell protein and expressed as mean ± SD of 3 independent experiments *P<0.01 vs DLPC, **P<0.001 vs DLPC.
(TIF)

Figure S2 **ADP stimulates autophagy.** HepG2 cells were serum-starved (Control) (**A**) or treated with 100 µM ADP (**B**) in serum-free DMEM media for 4 h. Cells were fixed and permeabilized and then apoA-I and LC3 were detected by indirect immunofluorescence using confocal microscopy. Original images of representative micrographs at 100× magnification from 2 independent experiments performed in quadruplicate are shown.
(TIF)

Figure S3 **Effect of autophagy inhibitors on cellular LC3-II.** HepG2 cells were pre-treated with 50 µM chloroquine, 5 mM 3-methyladenine (3-MA), 10 µM wortmannin or 10 µM U1026 for 30 min±100 µM ADP for 4 h in serum-free DMEM media. Cell lysates were immunoblotted for LC3. Histograms represent band densitometry analysis of LC3-II normalized to β-actin and expressed as percent change ± SD of 3 independent experiments. *P<0.05 vs Control and **P<0.01 vs Control.
(TIF)

Figure S4 **P2Y_13_ knockdown inhibits the ADP-dependent stimulation in autophagy.** HepG2 cells were transfected with either negative control (si-Ctrl) or P2Y_13_ siRNA (si-P2Y13) and incubated for 48 h. Cells were then incubated with ADP (100 µM) for 4 h in DMEM serum-free media. Cell lysates were immunoblotted for LC3 and blots are representative of 3 independent experiments.
(TIF)

Figure S5 **Reducing P2Y_13_ expression augments insulin receptor signaling.** HepG2 cells were transfected with either negative control (si-Ctrl) or P2Y_13_ siRNA (si-P2Y13) and incubated for 48 h. Cells were then pre-incubated with ADP (100 µM) for 5 min. and then with human insulin (100 nM) for 5 min in DMEM serum-free media. Cell lysates were immunoblotted for phosphorylated Akt (Ser473). Histograms represent densitometry analysis of p-Akt normalized to β-actin and expressed as mean percent change ± SD for 2 independent experiments.*P<0.05 vs si-Ctrl, **P<0.01 vs si- P2Y13.
(TIF)

Figure S6 **P2Y_13_ overexpression blocks insulin receptor signaling.** HepG2 cells were transfected with either a control pCMV plasmid (pCMV) or a pCMV plasmid expressing human P2Y_13_ (pCMV-P2Y13). Cell lysates were collected 48 h after transfection and immunoblotted for P2Y_13_ to measure protein overexpression (**inset, panel A**). Cells were then treated with human insulin (100 nM) for 5 min in DMEM serum-free media. Cell lysates were immunoblotted for insulin receptor (p-IR-β) (**A**) and phosphorylated Akt (Ser473) (**B**). Histograms represent densitometry analysis normalized to β-actin and are expressed as mean percent change ± SD for 3 independent experiments. (**A**)*P<0.001 vs pCMV and **P<0.01 vs pCMV+Insulin. (**B**) *P<0.001 vs pCMV and ** P<0.05 vs pCMV+Insulin.
(TIF)

Figure S7 **Reducing P2Y_13_ expression had no effect on the phosphorylation of mTOR.** HepG2 cells were transfected with either a negative control (si-ctrl) or a siRNA against human P2Y_13_. Cell lysates were collected 48 h after transfection and immunoblotted for phosphorylated Akt (Ser473) and phosphorylated mTOR (Ser2448). Histograms represent densitometry analysis of p-Akt and p-mTOR normalized to β-actin and expressed as mean ± SD of 3 independent experiments. *P<0.001 vs si-Ctrl.
(TIF)

Figure S8 **ADP increases mTOR phosphorylation.** HepG2 cells were treated with 100 µM ADP or 250 nM rapamycin for 4 h. Cell lysates were immunoblotted for phosphorylated mTOR (Ser2448). Histograms represent densitometry analysis of p-mTOR normalized to β-actin and expressed as mean percent change ± SD of 3 independent experiments. *P<0.05 vs control.
(TIF)
