# Supplementary figures and images for: Extracellular Nucleotides Inhibit Insulin Receptor Signaling, Stimulate Autophagy and Control Lipoprotein Secretion
Source: PLoS One. 2012 May 10;7(5):e36916. doi: 10.1371/journal.pone.0036916 (PMC3349634; doi:10.1371/journal.pone.0036916)

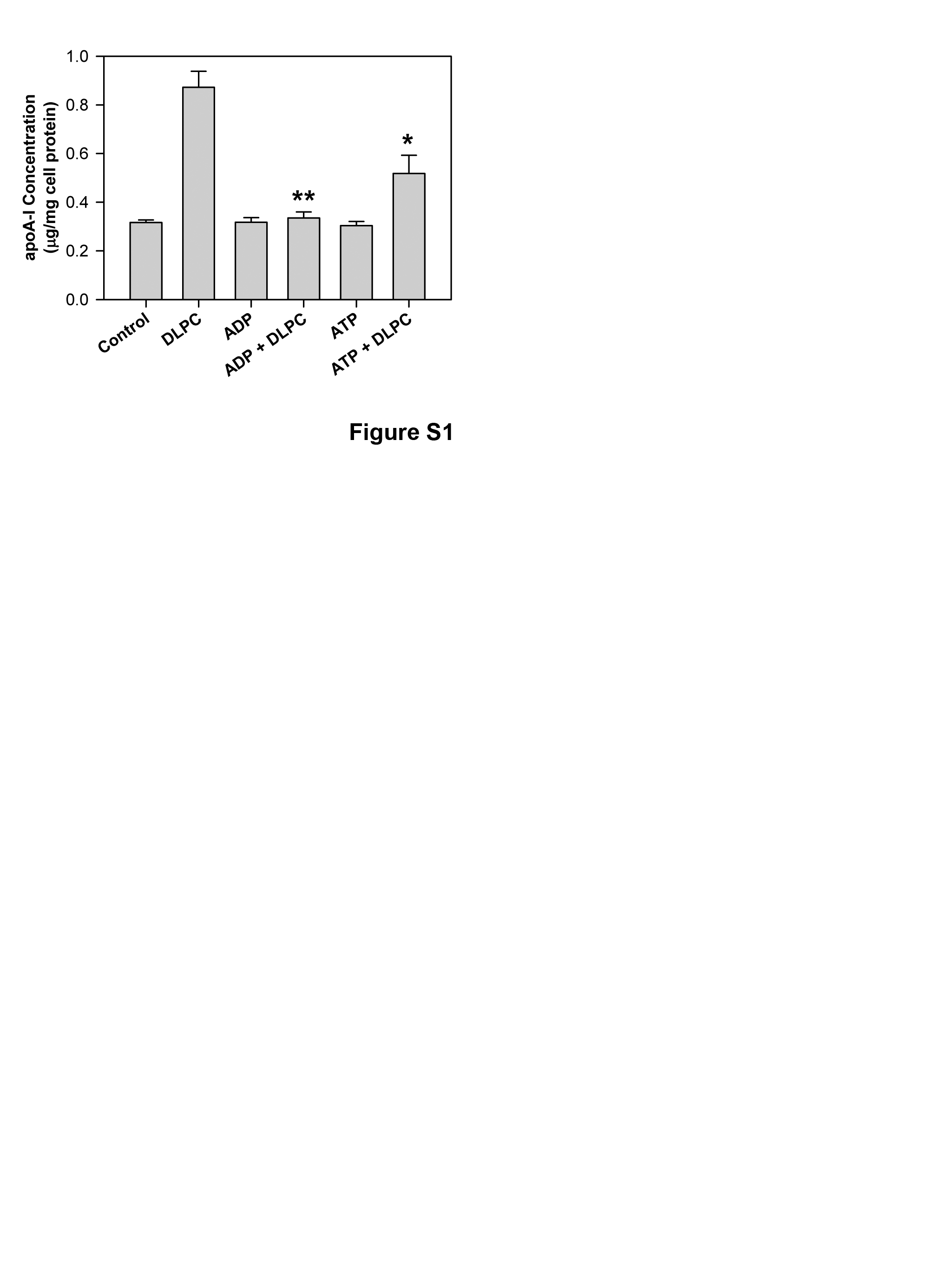

Supplement: Figure S1 — ATP decrease apoA-I levels in the media at 24 h. (A) HepG2 cells were pre-treated with adenosine diphosphate (ADP) or adenosine triphosphate (ATP) (100 µM) for 30 min. and then incubated with 12 µM DLPC in serum-free DMEM media. Conditioned media was collected after 24 h treatment and apoA-I concentration was quantified by ELISA. ApoA-I concentration in the media is normalized to total cell protein and expressed as mean ± SD of 3 independent experiments *P<0.01 vs DLPC, **P<0.001 vs DLPC. (TIF) [file pone.0036916.s001.tif]

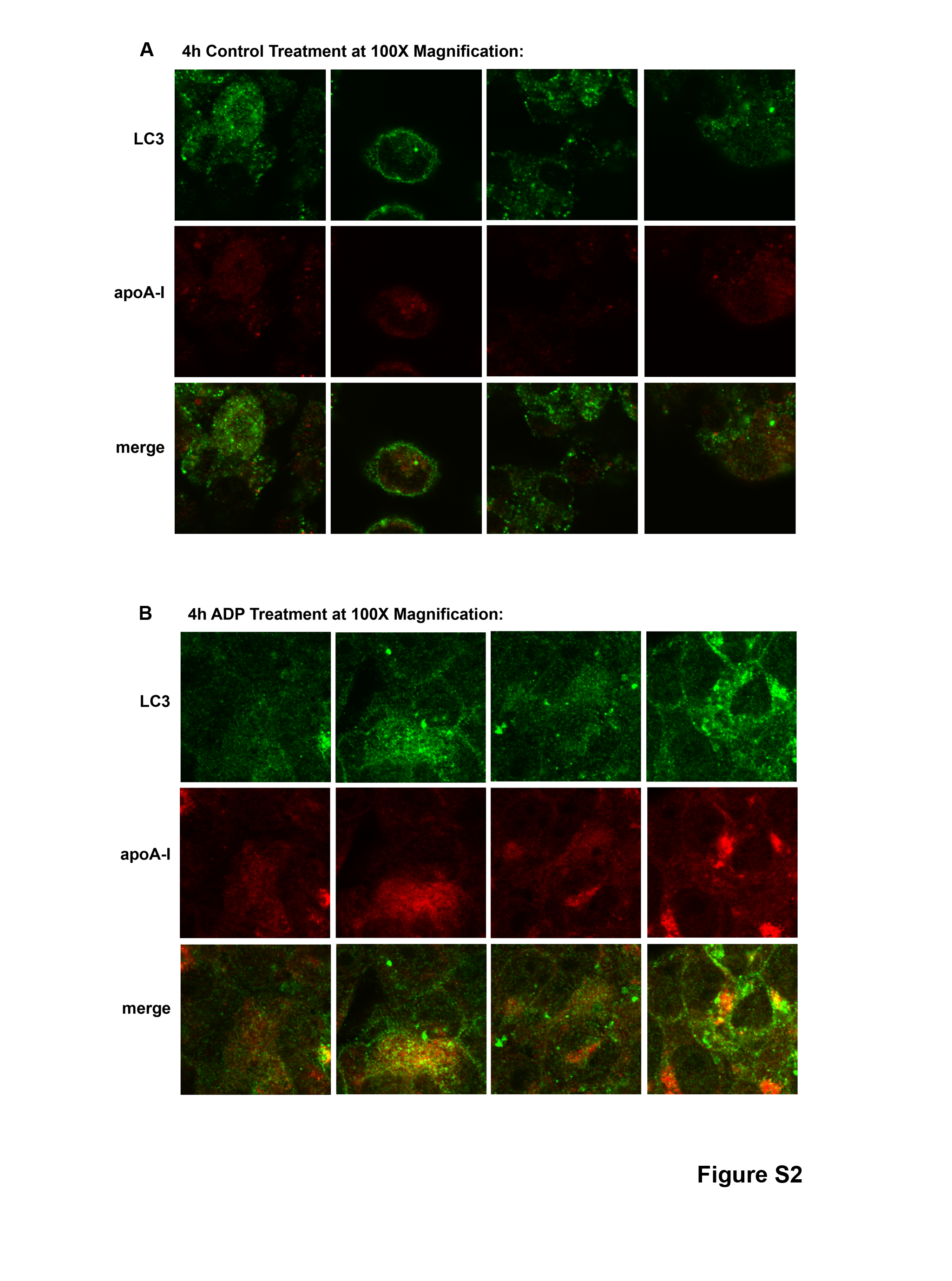

Supplement: Figure S2 — ADP stimulates autophagy. HepG2 cells were serum-starved (Control) (A) or treated with 100 µM ADP (B) in serum-free DMEM media for 4 h. Cells were fixed and permeabilized and then apoA-I and LC3 were detected by indirect immunofluorescence using confocal microscopy. Original images of representative micrographs at 100× magnification from 2 independent experiments performed in quadruplicate are shown. (TIF) [file pone.0036916.s002.tif]

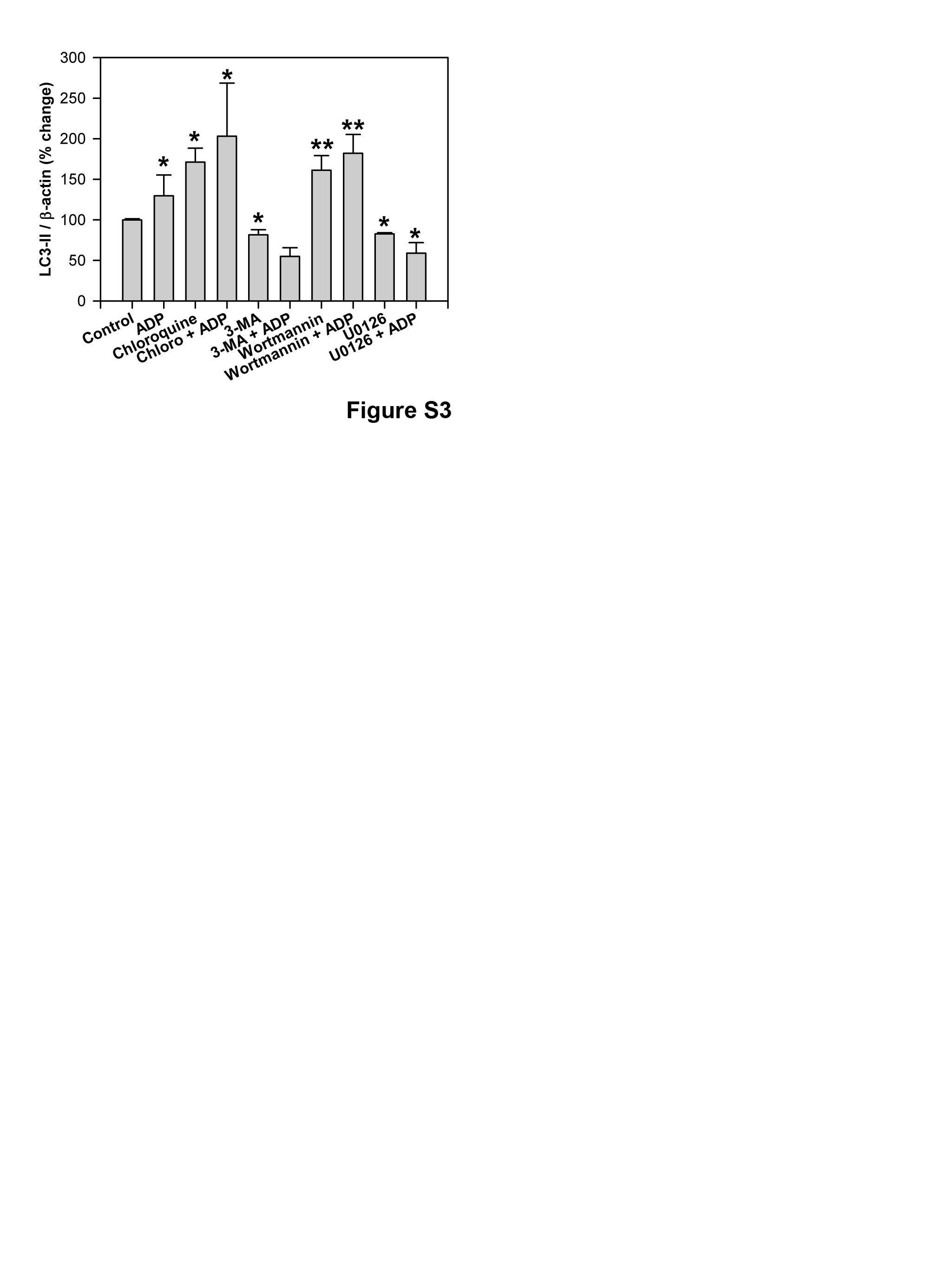

Supplement: Figure S3 — Effect of autophagy inhibitors on cellular LC3-II. HepG2 cells were pre-treated with 50 µM chloroquine, 5 mM 3-methyladenine (3-MA), 10 µM wortmannin or 10 µM U1026 for 30 min±100 µM ADP for 4 h in serum-free DMEM media. Cell lysates were immunoblotted for LC3. Histograms represent band densitometry analysis of LC3-II normalized to β-actin and expressed as percent change ± SD of 3 independent experiments. *P<0.05 vs Control and **P<0.01 vs Control. (TIF) [file pone.0036916.s003.tif]

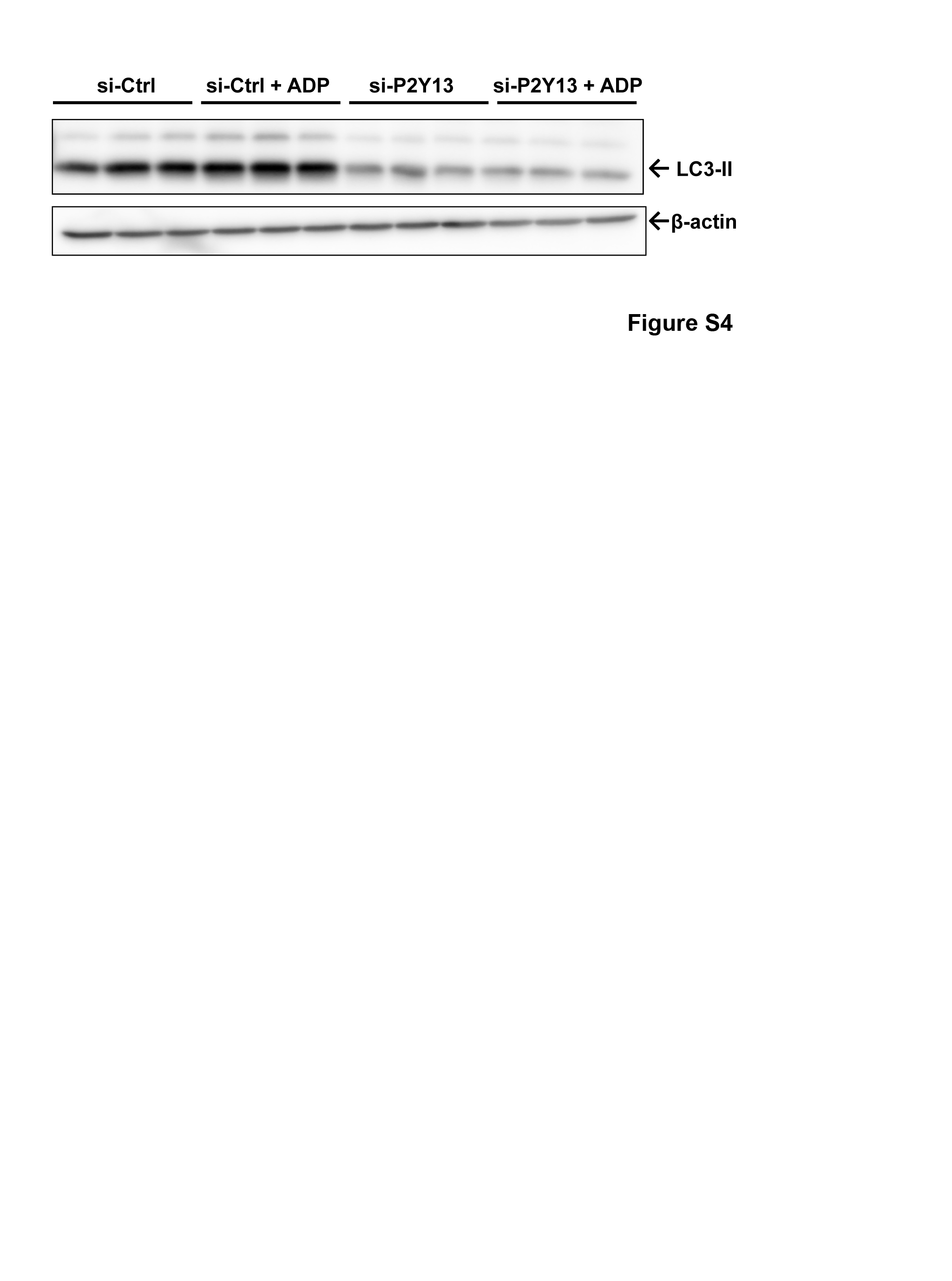

Supplement: Figure S4 — P2Y13 knockdown inhibits the ADP-dependent stimulation in autophagy. HepG2 cells were transfected with either negative control (si-Ctrl) or P2Y13 siRNA (si-P2Y13) and incubated for 48 h. Cells were then incubated with ADP (100 µM) for 4 h in DMEM serum-free media. Cell lysates were immunoblotted for LC3 and blots are representative of 3 independent experiments. (TIF) [file pone.0036916.s004.tif]

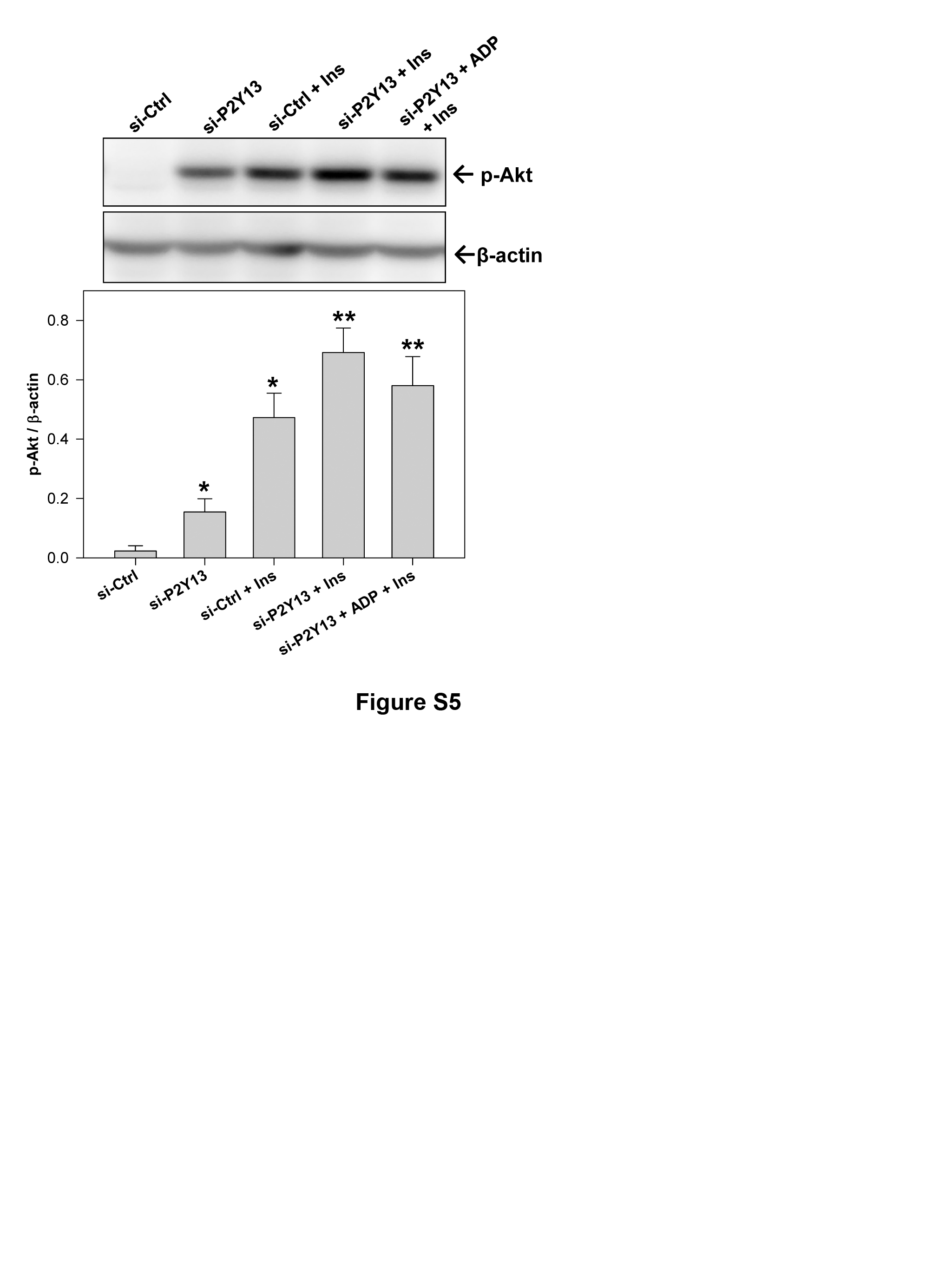

Supplement: Figure S5 — Reducing P2Y13 expression augments insulin receptor signaling. HepG2 cells were transfected with either negative control (si-Ctrl) or P2Y13 siRNA (si-P2Y13) and incubated for 48 h. Cells were then pre-incubated with ADP (100 µM) for 5 min. and then with human insulin (100 nM) for 5 min in DMEM serum-free media. Cell lysates were immunoblotted for phosphorylated Akt (Ser473). Histograms represent densitometry analysis of p-Akt normalized to β-actin and expressed as mean percent change ± SD for 2 independent experiments.*P<0.05 vs si-Ctrl, **P<0.01 vs si- P2Y13. (TIF) [file pone.0036916.s005.tif]

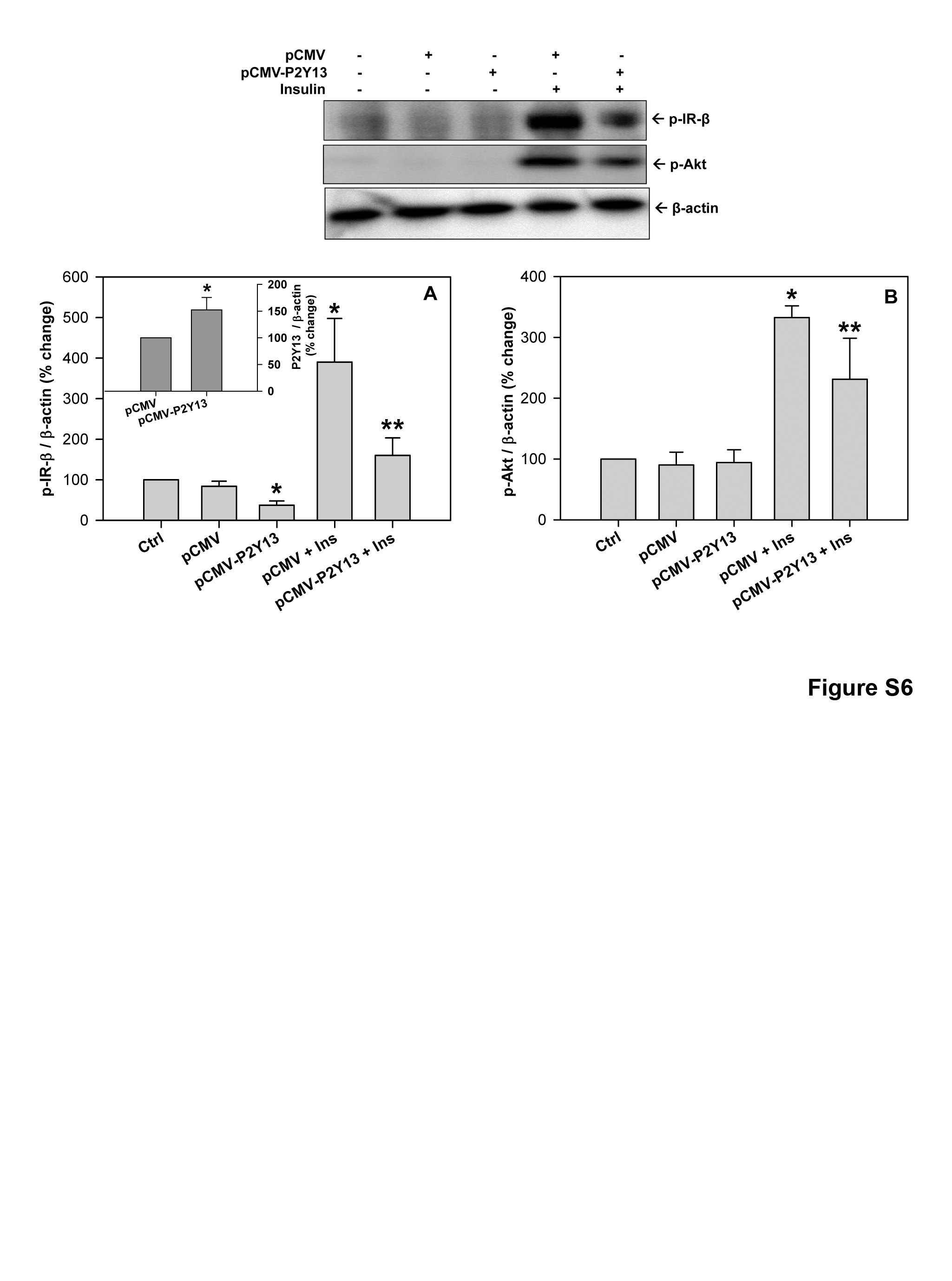

Supplement: Figure S6 — P2Y13 overexpression blocks insulin receptor signaling. HepG2 cells were transfected with either a control pCMV plasmid (pCMV) or a pCMV plasmid expressing human P2Y13 (pCMV-P2Y13). Cell lysates were collected 48 h after transfection and immunoblotted for P2Y13 to measure protein overexpression (inset, panel A). Cells were then treated with human insulin (100 nM) for 5 min in DMEM serum-free media. Cell lysates were immunoblotted for insulin receptor (p-IR-β) (A) and phosphorylated Akt (Ser473) (B). Histograms represent densitometry analysis normalized to β-actin and are expressed as mean percent change ± SD for 3 independent experiments. (A)*P<0.001 vs pCMV and **P<0.01 vs pCMV+Insulin. (B) *P<0.001 vs pCMV and ** P<0.05 vs pCMV+Insulin. (TIF) [file pone.0036916.s006.tif]

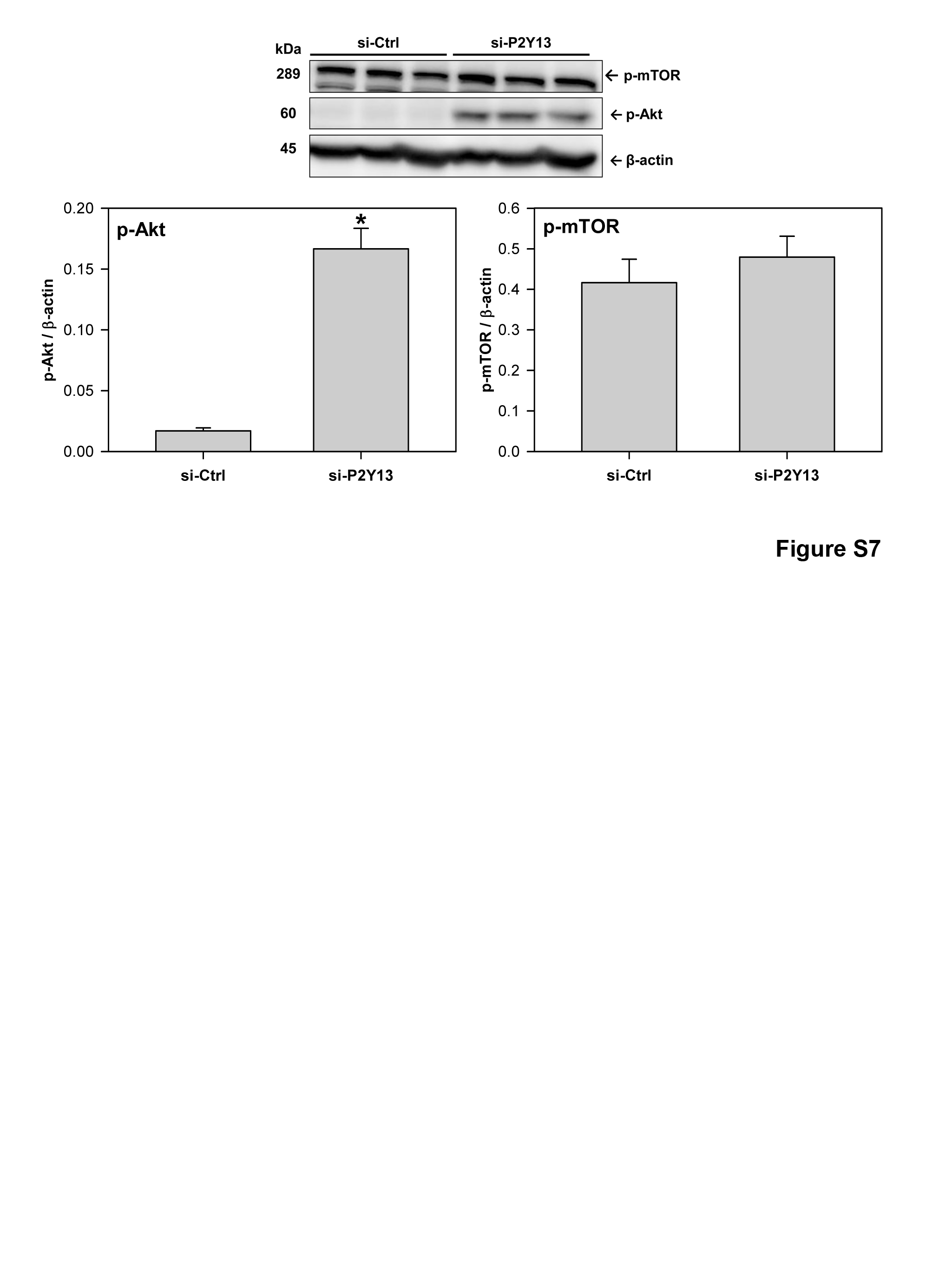

Supplement: Figure S7 — Reducing P2Y13 expression had no effect on the phosphorylation of mTOR. HepG2 cells were transfected with either a negative control (si-ctrl) or a siRNA against human P2Y13. Cell lysates were collected 48 h after transfection and immunoblotted for phosphorylated Akt (Ser473) and phosphorylated mTOR (Ser2448). Histograms represent densitometry analysis of p-Akt and p-mTOR normalized to β-actin and expressed as mean ± SD of 3 independent experiments. *P<0.001 vs si-Ctrl. (TIF) [file pone.0036916.s007.tif]

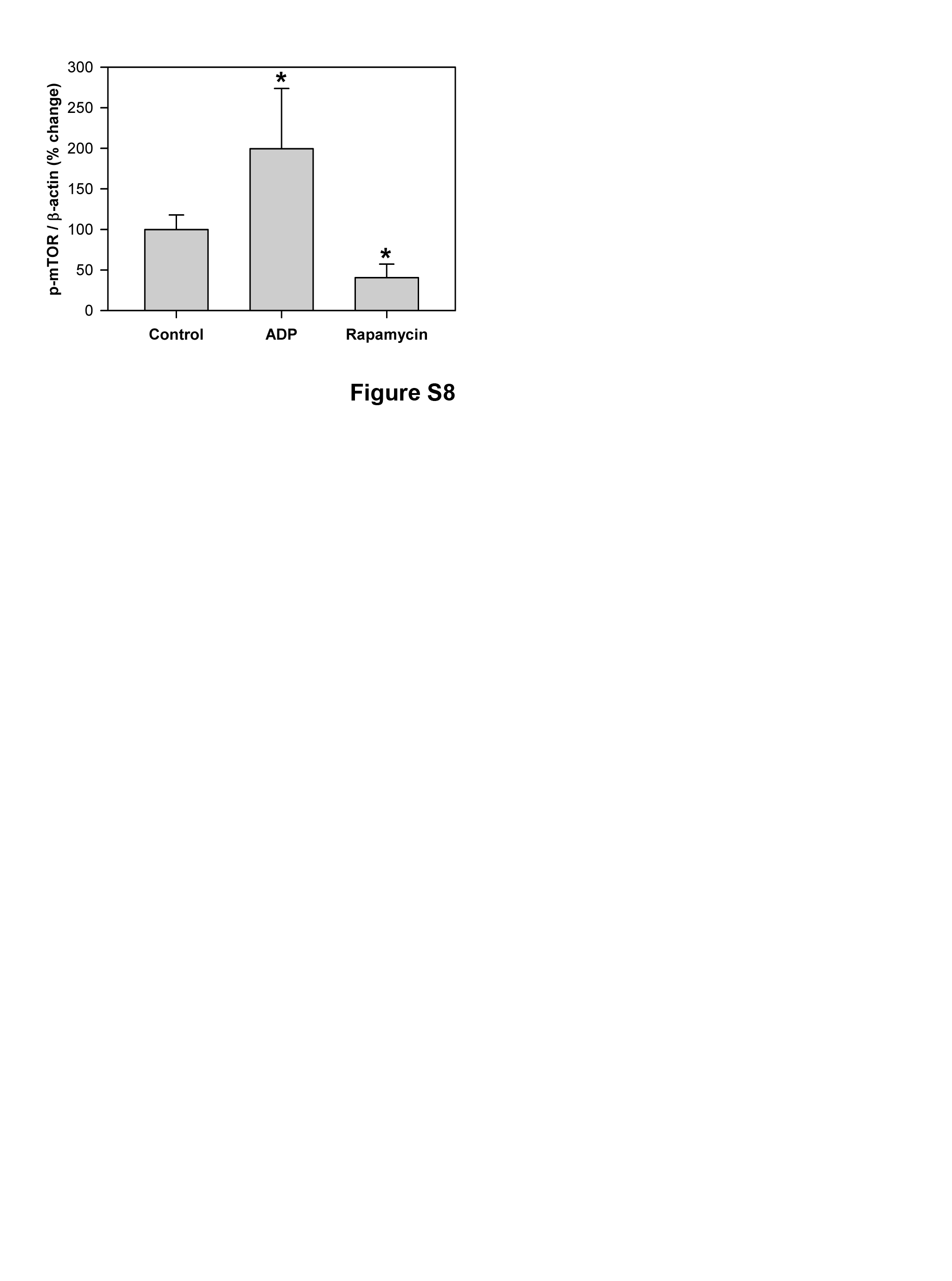

Supplement: Figure S8 — ADP increases mTOR phosphorylation. HepG2 cells were treated with 100 µM ADP or 250 nM rapamycin for 4 h. Cell lysates were immunoblotted for phosphorylated mTOR (Ser2448). Histograms represent densitometry analysis of p-mTOR normalized to β-actin and expressed as mean percent change ± SD of 3 independent experiments. *P<0.05 vs control. (TIF) [file pone.0036916.s008.tif]
